# *In silico* Investigation on the Inhibiting Role of Nicotine/Caffeine by Blocking the S Protein of SARS-CoV-2 Versus ACE2 Receptor

**DOI:** 10.3390/microorganisms8101600

**Published:** 2020-10-17

**Authors:** Saeedeh Mohammadi, Mohammad Heidarizadeh, Mehrnaz Entesari, Ayoub Esmailpour, Mohammad Esmailpour, Rasoul Moradi, Nader Sakhaee, Esmail Doustkhah

**Affiliations:** 1 Department of Physics, Shahid Rajaee Teacher Training University, Lavizan, Tehran 16788-15811, Iran; s.mohammadi427@gmail.com (S.M.); Esmailpour@sru.ac.ir (A.E.); 2 Department of Physics, Azarbaijan Shahid Madani University, Tabriz 53714-161, Iran; esmailpour@azaruniv.ac.ir; 3 Department of Microbiology, Faculty of Science, University of Maragheh, P.O. Box 55181-83111, Maragheh, Iran; hz.mohamad@yahoo.com; 4 Department of Genetic Engineering and Molecular Genetics, Zanjan University, Zanjan 45371-38791, Iran; mehrnaz.entesari@gmail.com; 5 Department of Chemical Engineering, School of Engineering and Applied Science, Khazar University, AZ1096 Baku, Azerbaijan; rasoulmoradi84@gmail.com; 6 Department of Chemistry, University of Illinois, Urbana, IL 61801, USA; nsakhaee@illinois.edu; 7 International Center for Materials Nanoarchitechtonics (WPI-MANA), National Institute for Materials Science (NIMS), 1-1 Namiki, Tsukuba, Ibaraki 305-0044, Japan

**Keywords:** anti-COVID-19, nicotine and caffeine, ACE2 human receptors

## Abstract

In this paper, we studied the *in silico* interaction of angiotensin-converting enzyme 2 (ACE2) human receptor with two bioactive compounds, i.e., nicotine and caffeine, via molecular dynamic (MD) simulations. The simulations reveal the efficient blocking of ACE2 by caffeine and nicotine in the exposure to the spike (S) protein of severe acute respiratory syndrome coronavirus 2 (SARS-CoV-2). We have selected the two most important active sites of ACE2-S protein, i.e., 6LZG and 6VW1, which are critically responsible in the interaction of S protein to the receptor and thus, we investigated their interaction with nicotine and caffeine through MD simulations. Caffeine and nicotine are interesting structures for interactions because of their similar structure to the candidate antiviral drugs. Our results reveal that caffeine or nicotine in a specific molar ratio to 6LZG shows a very strong interaction and indicate that caffeine is more efficient in the interaction with 6LZG and further blocking of this site against S protein binding. Further, we investigated the interaction of ACE2 receptor- S protein with nicotine or caffeine when mixed with candidate or approved antiviral drugs for SARS-CoV-2 therapy. Our MD simulations suggest that the combination of caffeine with ribavirin shows a stronger interaction with 6VW1, while in case of favipiravir+nicotine, 6LZG shows potent efficacy of these interaction, proposing the potent efficacy of these combinations for blocking ACE2 receptor against SARS-CoV-2.

## 1. Introduction

The rapid pandemic spread of a novel coronavirus disease 2019 (COVID-19), officially known as severe acute respiratory syndrome coronavirus 2 (SARS-CoV-2), is currently the most significant ongoing challenge to human health [1,2,3]. This new coronavirus can result in severe upper and lower respiratory infections, pneumonia, and kidney failure, and infected patients have a relatively high risk of mortality [4]. SARS-CoV-2 is the third highly pathogenic pneumonia coronavirus after SARS-CoV-1 and MERS-CoV (middle east respiratory syndrome-related coronavirus) to become a pandemic [5,6]. According to the World Health Organization (WHO), by 1 October 2020, over 34 million infections and 1,000,000 deaths have been reported [7]. COVID-19 has had a major impact on the normal daily lives of people throughout the world. Therefore, many researchers have been directed into his issue to overcome the pandemic outbreak of the COVID-19 virus. To fight this virus, three crucial and inevitable subjects are emphatically highlighted: developing an effective vaccine, discovery and/or synthesis of an effective drug to inhibit the progress of the virus in the infected body, and developing a fast and cheap technique with higher precision to diagnose infections.

To develop a new drug or repurpose one, it is essential to understand how a coronavirus infects the human body, how it attaches to cells, and how its progress in the host cells occurs. Looking at coronavirus structure, they are assembled as enveloped viruses with a positive-sense, single-stranded ribonucleic acid (RNA) genome [8,9,10]. SARS-CoV-2 contains four structural proteins including spike (S), envelope (E), membrane (M), and nucleocapsid (N) proteins [11,12]. It is well-known that SARS-CoV-2 engages its S protein to bind to the host cells through the protein-based receptor on the cells surface [13], called angiotensin converting enzyme 2 (ACE2). The structure of the complex between the SARS-CoV-2 S protein receptor binding domain (RBD) and the ACE2 receptor was determined to verify that the ACE2 receptor plays a notable role in mediating the entry of SARS-CoVs. ACE2 has an important role regulating the renin-angiotensin system (RAS), and it is confirmed that SARS-CoV infection reduces ACE2 expression and attenuates acute lung failure through blocking the renin–angiotensin pathway [14]. Finding a compound that can block the formation of this complex or disrupt in RBD-ACE2 complex has been suggested as a reasonable strategy to come up with a rational drug discovery for COVID-19 [15]. Although the development and discovery of a novel drug takes several years [16] with heavy costs, releasing a repurposed drug to market significantly lowers the consumed cost and time. Therefore, repurposing a drug with the above strategy has been recommended to rapidly deal with the current pandemic disease [17,18]. However, although several efforts are underway, there is no promising antiviral agent released for SARS-CoV-2. Therefore, we still need to review existing drugs in order to discover their antiviral properties [19].

Nicotine is a bioactive natural alkaloid that can be found in plants and acts as stimulant and inhibitor in the biological system of humans. Although nicotine contributes to the development of cardiovascular and pulmonary disease in the long term, it has an effective influence on the homeostasis of RAS by upregulating the angiotensin-converting enzyme (ACE)/angiotensin (ANG)-II/ANG II type 1 receptor axis and downregulating the compensatory ACE2/ANG-(1-7)/Mas receptor axis [20]. It is found that serum ACE activity experiences a significant increase immediately after smoking, and it requires at least 20 min to return to normal and reach to control levels [21]. In China, a systematic review and meta-analysis studies on 5960 currently smoking patients demonstrated that pharmaceutical nicotine should be considered as a potential treatment option in COVID-19 [22]. Also, other investigations have revealed the positive effects of nicotine as a potential treatment for COVID-19 in 6515 patients and has shown that hospitalized current smokers, compared to non-smokers, had higher recovery rates. This suggests a hypothesis that nicotine may be protective against severe COVID-19, which is biologically plausible and should be further investigated through clinical trials on nicotine as a drug candidate [23]. These clinical trials should test the effects of the smoking and nicotine on the risk of being infected with COVID-19 (NCT04429815). Based on the findings regarding nicotine that have been reported so far, we further investigated nicotine and caffeine (thanks to the similar chemical structure backbones of caffeine and nicotine) through in silico calculations to understand their potency blocking the S protein binding to the ACE2 receptor and, consequently, in suppressing COVID-19 progress in an infected body.

Although there are many reports of repurposing common antiviral agents for COVID-19 treatment, interaction between RBD-ACE2 complex and caffeine and/or nicotine through molecular dynamics (MD) and molecular docking methods have not yet been considered. Computational methods based on MD and molecular docking offer versatile and cost-effective tools for the preliminary examination of any potential drugs [24,25]. The mixed ligands of caffeine/nicotine with the currently proposed antiviral agents are our further focus of investigation, which we believe merit consideration. We hypothesized that there could be a significant S protein-ACE2 blocking potential activity through forming a complex between nicotine or caffeine with other current antiviral drugs (e.g., favipiravir, ribavirin, remdesivir, chloroquine, hydroxychloroquine, oseltamivir, and valganciclovir), so we investigated it through MD and molecular docking simulations. We also evaluated molars ratios of nicotine/caffeine to RBD/CTD-ACE2, solely and together with the other antiviral agents.

## 2. Methods

**Figure 1:**
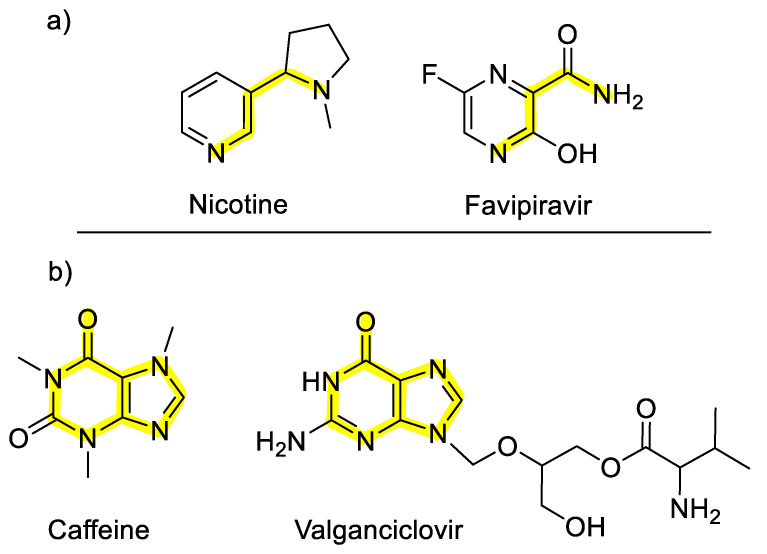
Comparing the chemical structure of (**a**) favipiravir with nicotine and (**b**) caffeine with valganciclovir; their similarities from chemical structure point of view are highlighted in yellow.

The interactions of two main compounds including nicotine and caffeine with the human ACE2 receptor -S protein (i.e., 6VW1 and 6LZG) were investigated. The chemical structure backbones of nicotine and caffeine resemble some candidate anti-COVID-19 drugs (i.e., favipiravir and valganciclovir; see Figure 1). Therefore, we aimed to investigate the possible interactions and potential blocking activity of these two molecules with the CTD/RBD-ACE2, which are the epitopes of S protein and the corresponding receptor responsible for COVID-19 cell binding, respectively. This blocking behavior is intensified when the compounds are mixed with other antiviral agents. Our focus will be directed toward the potency of nicotine (and caffeine) as COVID-19 antiviral drugs by blocking the ACE2 receptor mechanism.

### 2.1. Details of Structure ACE2 Receptors and SARS-CoV-2 Spike Protein Complexes

The several crystal structures of the complex between SARS-CoV-2 S protein and ACE2 receptor are recognized on the Protein Data Bank (PDB) (as PDB: 6LZG, 6VW1, 6M17) by X-ray diffraction and cryoelectron microscopy (cryo-EM). The 6LZG and 6VW1 structures as epitopes of the COVID-19 S protein were selected in this study. S protein in the 6LZG area has a non-chimeric nature, and the reported resolution of 6LZG is 2.50 Å, which is suitable for the generation of docking [26]. The crystal structure of the SARS-CoV-2 S protein RBD in complex with ACE2 (6VW1) has a resolution of 2.68 Å [27]. The SARS-CoV-2 S protein has two main functional subunits, including S1 and S2 subunit [26]. S1 can be further divided into an N-terminal domain (NTD) and a C-terminal domain (CTD), both of which can function as a receptor-binding entity [28]. The related crystal structure of the CTD SARS-CoV-2 S protein in complex with ACE2 is reported as 6LZG [26].

The ACE2 receptor has proteins that can be further divided into N-terminal peptidase M2 domain and C-terminal collectrin domain [26]. Thus, the complex of the ACE2 receptor and the S protein of SARS-CoV-2 are connected to each other through the RBD of the S protein and the ACE2 peptidase domain (PD). Hence, a strong binding between the RBD of the S protein and the CTD region of ACE2 receptor is to be expected [29]. The crystal structure of the SARS-CoV-2 S protein and the ACE2 receptor complex were visualized by SWISS-PdB viewer 4.1.0.22 from Plan-les-Oiiates/Geneva, Switzerland [30].

### 2.2. Selected Antiviral Agents

**Figure 2:**
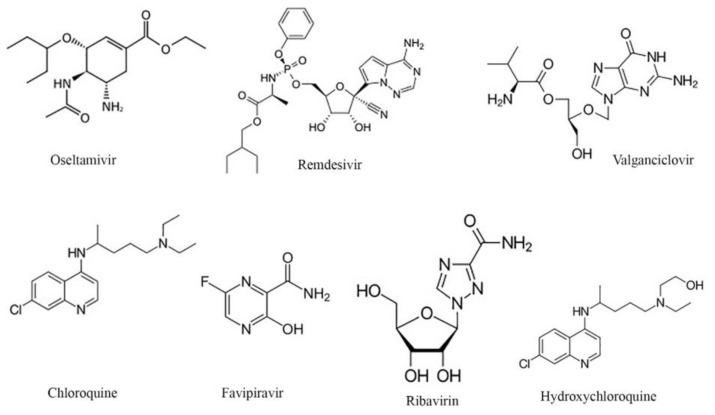
The phytochemical structures of antiviral drugs studied.

Several antiviral agents have been investigated for the treatment of COVID-19. Accordingly, we selected the seven antiviral drugs introduced by their chemical structure in Figure 2, and we selected the drug compounds shown in Table S1. We theoretically investigated the interaction of nicotine/caffeine with the RBD/CTD-ACE2 receptor (e.g., 6VW1 and 6LZG) when combined with these antiviral drugs to intensify the inhibition mechanism.

### 2.3. Molecular Dynamics Simulations

**Figure 3:**
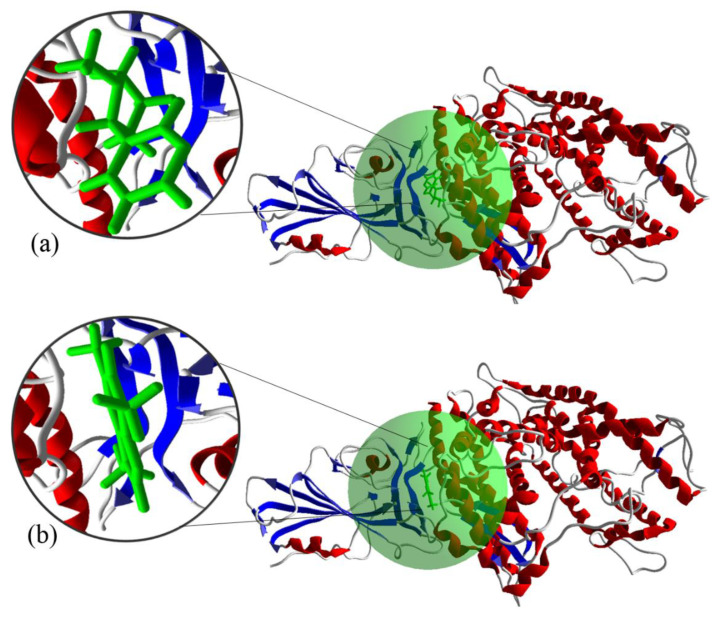
Interaction between ACE2/SARS-CoV-2-CTD (6LZG) complex with (**a**) nicotine and (**b**) caffeine. The green regions (and the corresponding magnified inlays) represent the active sites.

Molecular docking studies provide useful information in drug discovery before the execution of any costly empirical research. For example, Durdagi et al. used MD and molecular docking to analyze the binding interactions between novel fullerene inhibitors and human immunodeficiency virus type I aspartic protease (HIV-1 PR); these complexes, as HIV-1 PR inhibitors, possess higher bioactivity [31]. In the present work, our calculations based on molecular dynamic (MD) simulations were performed using Materials Studio 7 (Accelrys, Inc., San Diego, CA, USA). Furthermore, molecular docking has been utilized for more accurate and complimentary calculations regarding the binding conditions of the ligand (nicotine/caffeine-drugs) and RBD-ACE2 complex [32]. A structural representation of the interaction between ACE2/SARS-CoV-2-CTD and caffeine and nicotine is shown in Figure 3.

The structures of the RBD-ACE2 and ACE2-CTD complexes in bulk water were built under the periodic boundary conditions for MD simulations. The packing density was determined by an experimental bulk density of 1.0 $\left(g/{\mathrm{cm}}^{3}\right)$.

The structures of energy minimization were implemented using an alternating sequence of steepest descent [33] and conjugate gradient [34] optimizations, with 0.001 (kcal/mol) in the forcite module. For the intra and inter-molecular interactions and clusters calculations, the ab initio polymer consistence force fields (PCFF) was used [35,36,37]. The Andersen method [38] and the Berendsen method [39] were used to maintain the temperature (T=300 K) and the pressure, respectively. The simulation was performed using an integration time-step of 1.0 fs. All the dynamics runs were performed in a spline width of 1.0 Å and a buffer width of 0.5 Å.

**Figure 4:**
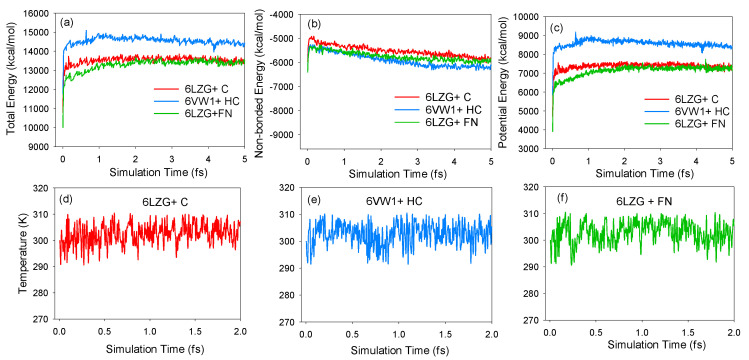
Energies and temperatures versus simulation time for (**a**) total, (**b**) non-bonded, and (**c**) potential energies. The plots show the temperatures for (**d**) CTD-ACE2+caffeine (6LZG+C), (**e**) RBD-ACE2+hydroxychloroquine+caffeine (6VW1+HC), and (**f**) CTD-ACE2+favipiravir+nicotine (6LZG+FN).

The non-bonding energy interactions were calculated within a cutoff distance of 15.5 Å. The long-range electrostatic interactions were computed using the Ewald summation method (ESM) [40]. It is generally believed that the system reaches equilibrium when the parameters such as energy and temperature fluctuations fall within the range of 5%. The calculation examples are represented in Figure 4. In Figure 4a–c, the total, non-bonding, and potential energies for CTD-ACE2+caffeine, RBD-ACE2+hydroxychloroquine+caffeine, and CTD-ACE2+favipiravir+nicotine, respectively, can be observed. Moreover, Figure 4d–f represents the temperature profile versus the simulated time for CTD-ACE2+caffeine, RBD-ACE2+hydroxychloroquine+caffeine, and CTD-ACE2+favipiravir+nicotine structures.

For further optimizing the membrane structure, we used an annealing simulation. Here, we raised the temperature from 300 K to 500 K and 100 ps for constant number of particles, pressure, and temperature (NPT) dynamics. In the next step, with a 250 ps NPT ensemble, we decreased the temperature from 300 K to 20 K. After relaxing the structures, NPT and constant number of particles, volume, and temperature (NVT) ensembles were performed for 500 ps.

### 2.4. Interaction Energy Calculation

By eliminating the water molecules from the optimized structures, we computed the interaction energy between the RBD/CTD-ACE2 complex and the drug ($E_{\mathrm{Int}\left(\mathrm{RD}\right)}$). Next, by removing the drug molecule, the energy of the isolated S protein-receptor, $E_{\mathrm{receptor}}$, was computed. Finally, by deleting the receptor from the S protein-receptor+drugs ensemble, the remaining energetic term for drugs, $E_{\mathrm{Drug}}$, was obtained.

The generalized interaction energy (IE) between the RBD/CTD-ACE2 and the drug that describes the system [41,42] is given by

(1) $$∆E_{Int\left(RD\right)}=E_{Recepter/Drug}-\left(E_{Receptor}-E_{Drug}\right).$$

### 2.5. Molecular Docking

It is suggested that a fast and economical tool (such as molecular docking) can be combined with molecular dynamics technology (precision but time consuming) and simulate more reliable and highly precise calculations in protein–ligand complexes. Moreover, for the fast screening of large libraries, docking can be efficiently utilized for exploring the conformations of the protein receptor, optimizing the structures of the final complexes, and calculating the accurate energies [43].

For targeting the interface of the S protein (in SARS-CoV-2) and the ACE2 receptor by a blocking agent, we evaluated the blocking potential of nicotine, caffeine, and their combined forms with other antiviral drugs. From the crystal structure reported by PDB for RBD-ACE2, the water molecules, 2-acetamido-2-deoxy-beta-D-glucopyranose, 1,2-ethanediol ligands, and the Zn and chloride ions were removed. Also, from crystal structure of ACE2/SARS-CoV-2-CTD, the water molecules, 2-acetamido-2-deoxy-beta-D-glucopyranose ligand, and the Zn ion were removed. The hydrogen atoms in these structures were enhanced in this study. We used the AutoDock v4.2 package (AutoDock is being developed and maintained in the Forli Laboratory, with support from the US National Institutes of Health) for the docking study [44,45]. Also, the charges of the molecules were applied. We selected a 60 × 60 × 60 Å grid box, and the distance between two grid points was set at 1.0 Å centering on the structures. In this paper, the rigid structure of the proteins was considered, so that, in this state, the drug is assumed to be fixed in shape. By using the Lamarckian genetic algorithm (LGA) [46], we performed molecular docking. In molecular docking through genetic algorithms (GA) [47], the particular arrangement of a ligand and a protein can be defined by a set of values describing the translation, orientation, and conformation of the ligand with respect to the protein. In the GA, there are some variable states in the ligand’s state, and each variable state can correspond to a specific gene. Random pairs of individuals are mated using a process of crossover, in which new individuals inherit genes from either parent. In addition, some offspring undergo random mutation, in which one gene change to a random extent [46]. Thigh profile was achieved under the following conditions: an initial population of 150 randomly placed individuals and a maximum number of 2.5 × 106 energy evaluations, a maximum number of 27,000 generations, a mutation rate of 0.02, a crossover rate of 0.80, and an elitism value of 1.

## 3. Results and Discussion

Here, we present our numerical results from calculating the interaction between RBD-ACE2 and CTD-ACE2 and antiviral drugs with nicotine or caffeine. We highlight two ligands (i.e., caffeine and nicotine) that we believe may be of special interest for experimental evaluation.

### 3.1. Molar Ratios of Nicotine and Caffeine to RBD/CTD-ACE2 Receptor

**Figure 5:**
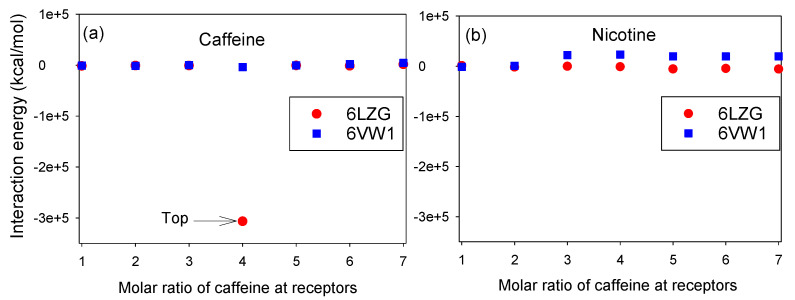
The interaction energies (IEs) for different ratios of (**a**) caffeine and (**b**) nicotine structures with 6LZG and 6VW1.

In this section, we studied the effect of different molar ratios of caffeine/nicotine on the IE complex of 6LZG/6VW1+caffeine/nicotine. Our results show that using different molar rates of caffeine and nicotine led to a change of IE in both RBD-ACE2 and ACE2/SARS-CoV-2-CTD. Based on the MD simulations results of the ACE2-S protein, we predicted the binding between ligands and proteins with low IE. As shown in Figure 5a, the existence of 4 mol of caffeine in the ACE2/SARS-CoV-2-CTD complex showed the lowest IE complex. As shown this figure, the top compound that could target the binding interface between S protein and ACE2 is 4 mol of caffeine. In the case of nicotine, there are different IE trends in both 6VW1 and 6LZG, but the trends slightly change in comparison with caffeine, which are illustrated in Figure 5b. Overall, compared to nicotine, caffeine has a stronger interaction with the RBD-ACE2 receptor, especially with an ACE2/SARS-CoV-2-CTD complex. We can see that 4 mol of caffeine and the 6LZG complex do not behave similarly to 6VW1. It is therefore necessary to study how caffeine is connected with the amino acids of 6LZG and 6VW1. In Section 3.5, we will describe how amino acids are connected to caffeine in detail.

### 3.2. Interaction Energy Complex of RBD-ACE2 and CTD-ACE2 with Nicotine or Caffeine and Drugs

**Figure 6:**
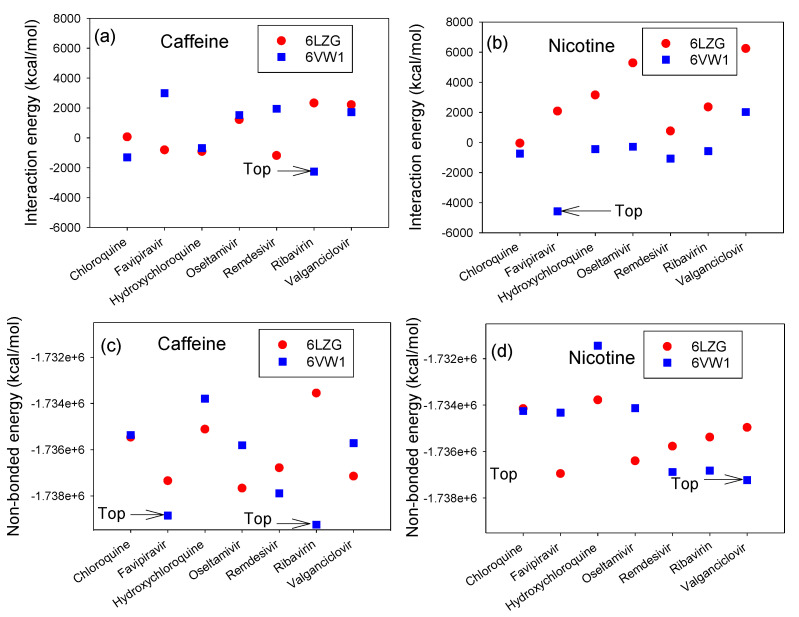
The interaction and non-bonded energies for drug and caffeine/nicotine structures with 6VW1 (RBD-ACE2) and 6LZG (CTD-ACE2). (**a**,**b**) The interaction energies caffeine and nicotine, (**c**,**d**) the non-bonded energies caffeine and nicotine, respectively.

**Figure 7:**
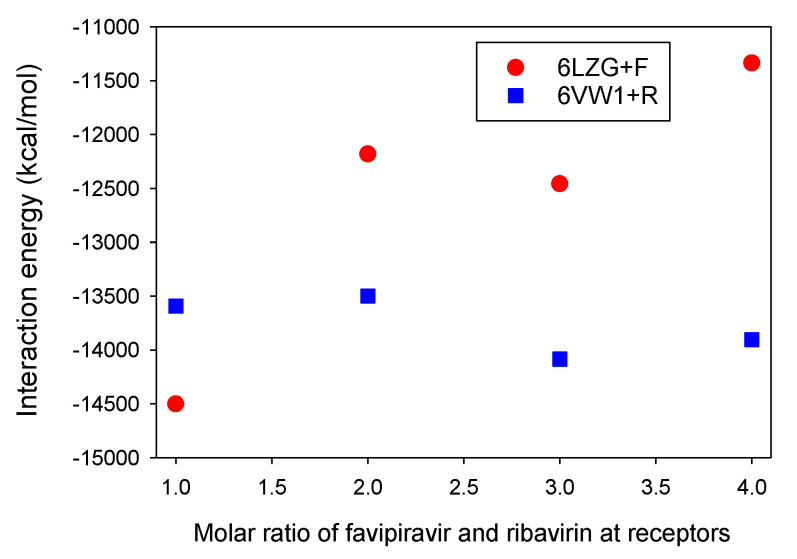
The bond and van der Waals energies for drug and caffeine/nicotine structures with 6VW1 (RBD-ACE2) and 6LZG (CTD-ACE2). (**a**,**b**) The bond energies caffeine and nicotine, (**c**,**d**) the van der Waals energies caffeine and nicotine, respectively.

In this paper, we set the structures energies as follows:

(2) $$∆E_{B}=E_{Bond}^{Final}-E_{Bond}^{Reference}$$

(3) $$∆E_{NB}=E_{Non-Bond}^{Final}-E_{Non-Bond}^{Reference}$$

(4) $$∆E_{vdW}=E_{vanderWaals}^{Final}-E_{vanderWaals}^{Reference}$$

where $∆E_{B}$ and $∆E_{\mathrm{NB}}$ refer to the systems bond and non-bonded intra-molecular interaction energies, respectively. $E_{\mathrm{Bond}}^{\mathrm{Final}}$ and $E_{\mathrm{Non}-\mathrm{Bond}}^{\mathrm{Final}}$ describe the bond and non-bonded energies of the final systems, respectively, and $E_{\mathrm{Bond}}^{\mathrm{Reference}}$ and $E_{\mathrm{Non}-\mathrm{Bond}}^{\mathrm{Reference}}$ are the bond and non-bonded energies of the reference receptors and drugs, respectively. $∆E_{\mathrm{vdW}}$ is the van der Waals energy, and $E_{\mathrm{vanderWaals}}^{\mathrm{Final}}$ and $E_{\mathrm{vanderWaals}}^{\mathrm{Reference}}$ refer to the reference and final energies of the systems, respectively. The interaction energies, i.e., $∆E_{B}$, $∆E_{\mathrm{NB}}$ and $∆E_{\mathrm{vdW}}$, of antiviral drugs with nicotine or caffeine on the RBD/CTD-ACE2 complex were evaluated (Figure 6 and Figure 7). According to the results, the system with low IE values is more stable (i.e., when the IE values are more negative, the interaction force between RBD-ACE2 or CTD-ACE2 with the ligand is noteworthy). It is observed that the IE values of the RBD/CTD-ACE2+caffeine complex and RBD/CTD-ACE2+nicotine were found to be more stable. Also, an improvement in the IE values was observed when the antiviral drugs were combined with either nicotine or caffeine, showing that the antiviral drugs enhance the tendency of a significant interaction with the ACE2 receptor and the S protein. Creating adhesion between the receptors and ligands causes the breaking of the non-bonded intramolecular bonds in the receptor protein, and as a result, this causes a free energy penalty. Therefore, the competition between the inter- and intramolecular interactions are critical in determining the successful adhesion of ACE2 with ligand(s) (e.g., caffeine or nicotine). As illustrated in Figure 6a,b, of the 28 possible interactions (see Table S1), the IEs of favipiravir+nicotine with CTD-ACE2 and ribavirin+caffeine with RBD/CTD-ACE2 are lower than those of the other interactions. There are several possible reasons for this observation: a high contact area between the S protein-ACE2 and the combined ligands; the nucleosidic nature of favipiravir and ribavirin, which allows for better adhesion; or a high amount of hydrogen bonding can be formed between the protein and the combined ligands [48]. Notably, the matchable hydrophobicity and the complementarity shape of the receptor protein and ligands play a pivotal role in determining the blocking activity of the ligand [49].

When the difference between $∆E_{B}$ and $∆E_{\mathrm{NB}}$ decreases, by increasing the $∆E_{B}$ and decreasing the $∆E_{\mathrm{NB}}$, the receptor has a tendency to agglomerate. These results are due to the short distance of binding force between the receptors and/or creating hydrogen bonds between the receptors and drugs. Therefore, the most effective combinations are favipiravir with nicotine in exposure to CTD-ACE2, valganciclovir with nicotine in exposure to RBD-ACE2, favipiravir with caffeine in exposure to RBD-ACE2, and ribavirin with caffeine in exposure to RBD-ACE2, as illustrated in Figure 6c,d.

Figure 7a,b reveals that, in case of the bonding and van der Waals interactions, in exploring the combination of caffeine/nicotine with antiviral drugs in exposure to ACE2-S protein, the combination of favipiravir with caffeine has a prevailing interaction with RBD-ACE2. In the case of nicotine, valganciclovir has a better combination result in interaction strength during exposure to RBD-ACE2. Therefore, as shown in Figure 7c,d, the most efficient compounds are valganciclovir with nicotine versus RBD-ACE2, remdesivir with nicotine versus RBD-ACE2, oseltamivir with nicotine versus CTD-ACE2, favipiravir and caffeine versus RBD-ACE2, and finally ribavirin and caffeine versus RBD-ACE2.

### 3.3. Molar Ratios of Favipiravir and Ribavirin to S Protein-ACE2 Receptor

**Figure 8:**
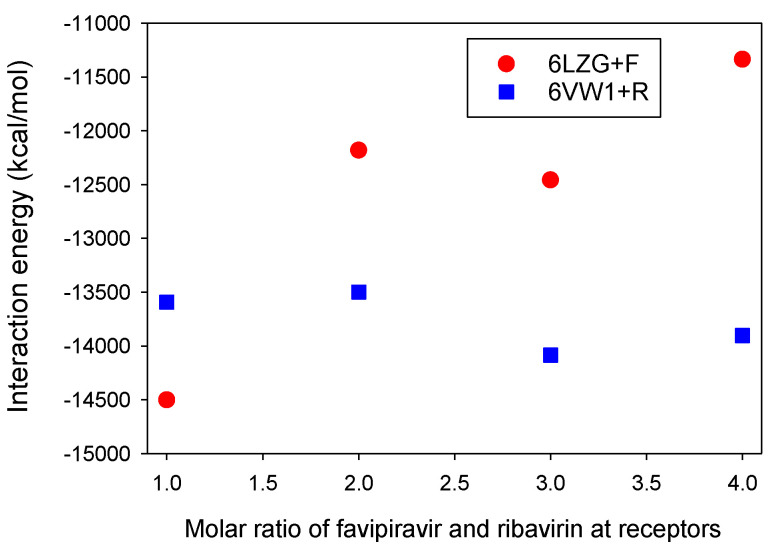
The interaction energies for different ratios of the structures of favipiravir and ribavirin with ACE2/SARS-CoV-2-CTD (6LZG) and RBD-ACE2 (6VW1). The 6LZG+F and 6VW1+R display 6LZG+favipiravir and 6VW1+ribavirin, respectively.

We have reported the determination of molar ratio resonance assignments for the S protein-ACE2 in complex with ligands, e.g., favipiravir and ribavirin. As shown in Figure 8, the contribution of RBD-ACE2 dynamics can be computed for different molar ratios for favipiravir and ribavirin in the absence of nicotine and caffeine. It is observed that one mol of favipiravir+CTD-ACE2, in comparison with ribavirin+RBD-ACE2, has a strong interaction.

### 3.4. Interaction Energy of ACE2 with Ranked Compounds

Here, in the absence of an S protein, we computed the interactions of the ACE2 receptor with the candidate drugs (e.g., favipiravir, ribavirin, and remdesivir). We aimed to observe the effect of blocking ACE2 without the S protein. Thus, the best conformations were selected on the basis of interaction energy or binding affinity between two interacting systems. Therefore, we selected the top three combinations that respond well in Section 3.2, such as ribavirin, favipiravir, and remdesivir with caffeine. Our MD simulations show that the interaction energies for ACE2+ribavirin+caffeine, ACE2+favipiravir+caffeine, and ACE2+remdesivir+caffeine were found as follows: 469.61, 843.31, and −396.68 (kcal/mol), respectively. As a result, ACE2+remdesivir+caffeine exhibited a very strong interaction in the absence of the S protein.

### 3.5. Molecular Docking of the S Protein-ACE2 with Compounds

In this section, we present our molecular docking results from calculating the interaction of RBD-ACE2 and CTD-ACE2 with drug compounds and nicotine/caffeine. Basically, AutoDock computes the best binding mode using binding free energy evaluation. Also, AutoDock calculates the binding free energy using inter-molecular energy (which consists of van der Waals energy, hydrogen bonding energy, desolation energy, and electrostatic energy), internal energy of ligand, and torsional free energy [50]. The binding energy is due to the energy contributed by different amino acids or residues around the cavity of target protein on interaction with the screened molecule. These residues contribute energy is due to different interactions like hydrogen bonding, van der Waals, electrostatic interactions, π-π stacking, etc. [51].

As the binding of the S protein to ACE2 is undesirable, it is preferable to diminish the ligand–interface interactions that may bridge, and therefore stabilize, the interaction between the S protein and the ACE2 receptor. The strongest binding affinity, initial repurposing may be better suited to nicotine+favipiravir+ CTD-ACE2 and caffeine+ribavirin+6VW1 (Figure S1). The molecular docking calculations illustrates that the nicotine+favipiravir+ CTD-ACE2 and caffeine+ribavirin+ RBD-ACE2, equal to −7.13 and −6.76 (kcal/mol), respectively, have the lowest binding free energy and, hence, the strongest interactions. It seems that variation in the binding free energies occurs due to the difference in the hydrophobic interactions and hydrogen bonding formation between RBD/CTD-ACE2′s amino acid residues with drugs and nicotine/caffeine. The system stabilization can occur by lowering the binding free energy for the most stable conformation. The nicotine+favipiravir+ CTD-ACE2 forms hydrophobic interactions with nine amino acids, which are Ala386, Ala387, Arg393, Asn33, Gln388, Glu37, His34, Phe390, and Pro389, from the target ACE2 with nicotine and Arg403 and Tyr505 of the S protein. The hydrogen bonds created the interactions between the N groups of the nicotine with amino acid residues Arg403 and Tyr505, whereas residues Ala396, Asn394, Asn397, Asp206, Glu398, Gly395, and Lys362 of ACE2 receptor have hydrophobic interactions with favipiravir (see Table 1 and Figures S2 and S3). Also, for caffeine+ribavirin+RBD-ACE2, there are hydrophobic interactions between six amino acids (e.g., Ala386, Ala387, Arg393, Asp355, Glu37, Gly352 of ACE2) and ribavirin. There are other residues, such as Asp405, Gly502, Gly504, Tyr505, and Val503, of SARS-CoV-2 chimeric RBD that are connected with ribavirin. There are interactions between the hydrogen bonding groups of the ribavirin with amino acid residues (Lys353, Gly354, Asp350) that bind to ACE2 and amino acids, like Tyr505 or Gly504 of the S protein. The two hydrogen bonds formed with the amino acid residues are (a) the hydrogen atom of the O-H group with amino acids, including Gly504 and Lys353; and (b) the nitrogen atom of N-terminal of ribavirin with amino acids such as Asp350, Tyr505, and Gly354 (see Table 2 and Figures S4 and S5).

## 4. Conclusions

In summary, we theoretically evaluated (using MD simulations) the interactions of two crucial active sites of the S protein (i.e., 6LZG, 6VW1) when it is complexed with the ACE2 receptor using two accessible natural bioactive alkaloids, i.e., nicotine and caffeine. Then, the combination of nicotine and caffeine with antiviral drugs—including favipiravir, ribavirin, remdesivir, chloroquine, hydroxychloroquine, oseltamivir, and valganciclovir—were investigated as potential complimentary agents of the ACE2 receptor. The results of the MD simulations revealed a promising binding tendency in caffeine/nicotine with the ACE2 receptor; consequently, the blocking of the ACE2 receptor against SARS-CoV-2 can occur. The results indicate that the molar ratio of caffeine and nicotine with 6LZG and 6VW1 also has a major impact on the blocking activities of the ACE2 receptor. We have shown that, in the case of the 6VW1 complex, caffeine with favipiravir and ribavirin form a more permeant structure against SARS-CoV-2 in term of non-bonding interaction energy. Also, we show that the combination of nicotine and favipiravir in blocking 6LZG and the combination of caffeine and ribavirin in blocking 6VW1 were more successful. In conclusion, our results suggest that nicotine and caffeine compounds have the capability to interact with the S protein and ACE2 and interfere with their binding through blocking the active sites. The results might have significant applications in considering them as drug candidates in therapeutic treatment of SARS-CoV-2 infection.

## Figures and Tables

**Table 1:** Contact residues of the SARS-CoV-2 CTD-ACE2 interfacing with favipiravir and nicotine. The residues involved in the hydrogen and hydrophobic interactions between S protein and ACE2 with favipiravir+nicotine.

	Favipiravir		Nicotine	
	S protein	ACE2	S protein	ACE2
**Hydrogen bonding**		-	Arg403(N)-Tyr505(N)	-
**Hydrophoic interactions**		Ala396-Asn394-Asn397-Asp206-Glu398-Gly395-Lys362		Ala386-Ala387-Arg393-Asn33-Gln388-Glu37-His34-Phe390-Pro389

**Table 2:** Contact residues of the SARS-CoV-2 RBD-ACE2 interfacing with ribavirin and caffeine. Residues involved in the hydrogen and hydrophobic interactions between S protein and ACE2+ribavirin+caffeine.

	Ribavirin		Caffeine	
	S protein	ACE2	S protein	ACE2
**Hydrogen bonding**	Gly504(O, O-H)-Tyr505(N)	Lys353(O)-Asp350(NH_2_)-Gly354(N)	-	-
**Hydrophoic interactions**	Asp405-Gly502-Val503	Ala386-Ala387-Arg393-Asp355-Glu37-Gly352-Leu351-Met383-Phe356-Thr324		Arg393-Asn394- Asp350-Glu37-Gly352-Leu351-Leu391-Leu392-Phe40-Phe390

## Supplementary Materials

The following are available online at https://www.mdpi.com/2076-2607/8/10/1600/s1, Figure S1: Interaction between ACE2-RBD/CTD (6VW1, 6LZG) complex with (**a**) 6LZG+favipiravir+nicotine and (**b**) 6VW1+ribavirin+caffeine that green region and magnified inlays represent active sites; Figure S2: 2D representation of favipiravir binding mode with receptor binding site of SARS-CoV-2 S protein (6LZG+favipiravir+nicotine); Figure S3: 2D representation of nicotine binding mode with receptor binding site of SARS-CoV-2 S protein (6LZG+favipiravir+nicotine); Figure S4: 2D representation of ribavirin binding mode with receptor binding site of SARS-CoV-2 S protein (6VW1+ribavirin+caffeine); Figure S5: 2D representation of caffeine binding mode with receptor binding site of SARS-CoV-2 S protein (6VW1+ribavirin+caffeine); Table S1: Selected drug compounds with S protein (RBD) and ACE2 receptor.
