# Supplementary material for: In silico Investigation on the Inhibiting Role of Nicotine/Caffeine by Blocking the S Protein of SARS-CoV-2 Versus ACE2 Receptor
Source: Microorganisms. 2020 Oct 17;8(10):1600. doi: 10.3390/microorganisms8101600 (PMC7603175; doi:10.3390/microorganisms8101600)
Supplement: Supplementary file 1 [file microorganisms-08-01600-s001.pdf]

In silico investigation on potential blocking activity of nicotine and caffeine on the interaction of human ACE2 and SARS-CoV-2 spike protein

Saeedeh Mohammadi [1,2], Mohammad Heidarizadeh [3], Mehrnaz Entesari [4], Ayoub Esmailpour [1], Mohammad Esmailpour [2], Rasoul Moradi [5], Nader Sakhaee [6] and [*]Esmail Doustkhah [7]

[1] Department of Physics, Shahid Rajaee Teacher Training University, Lavizan, Tehran 16788-15811, Iran
[2] Department of Physics, Azarbaijan Shahid Madani University, Tabriz, Iran
[3] Department of Microbiology, Faculty of Science, University of Maragheh, PO Box 55181-83111, Maragheh, Iran
[4] Department of Genetic Engineering and Molecular Genetics, Zanjan University, Zanjan, Iran
[5] Department of Chemical Engineering, School of Engineering and Applied Science, Khazar University, Baku, Azerbaijan
[6] Department of Chemistry, University of Illinois, Urbana, IL 61801, USA
[7] International Center for Materials Nanoarchitechtonics (WPI-MANA), National Institute for Materials Science (NIMS), 1-1 Namiki, Tsukuba, Ibaraki 305-0044, Japan
* Correspondence: esmaildostkhah@gmail.com

Table S1: Selected drug compounds with S protein (RBD) and ACE2 receptor.

6LZG+favipiravir+caffeine	6LZG+favipiravir+nicotine
6LZG+ ribavirin+caffeine	6LZG+ ribavirin+ nicotine
6LZG+remdesivir+caffeine	6LZG+remdesivir+ nicotine
6LZG+chloroquine+caffeine	6LZG+chloroquine+ nicotine
6LZG+hydroxychloroquine+caffeine	6LZG+hydroxychloroquine+nicotine
6LZG+ oseltamivir+caffeine	6LZG+ oseltamivir+ nicotine
6LZG+valganciclovir+caffeine	6LZG+valganciclovir+nicotine
6VW1+favipiravir+caffeine	6VW1+favipiravir+nicotine
6VW1+ ribavirin+caffeine	6VW1+ ribavirin+ nicotine
6VW1+remdesivir+caffeine	6VW1+remdesivir+ nicotine
6VW1+chloroquine+caffeine	6VW1+chloroquine+ nicotine
6VW1+hydroxychloroquine+caffeine	6VW1+hydroxychloroquine+nicotine
6VW1+ oseltamivir+caffeine	6VW1+ oseltamivir+ nicotine
6VW1+valganciclovir+caffeine	6VW1+valganciclovir+nicotine

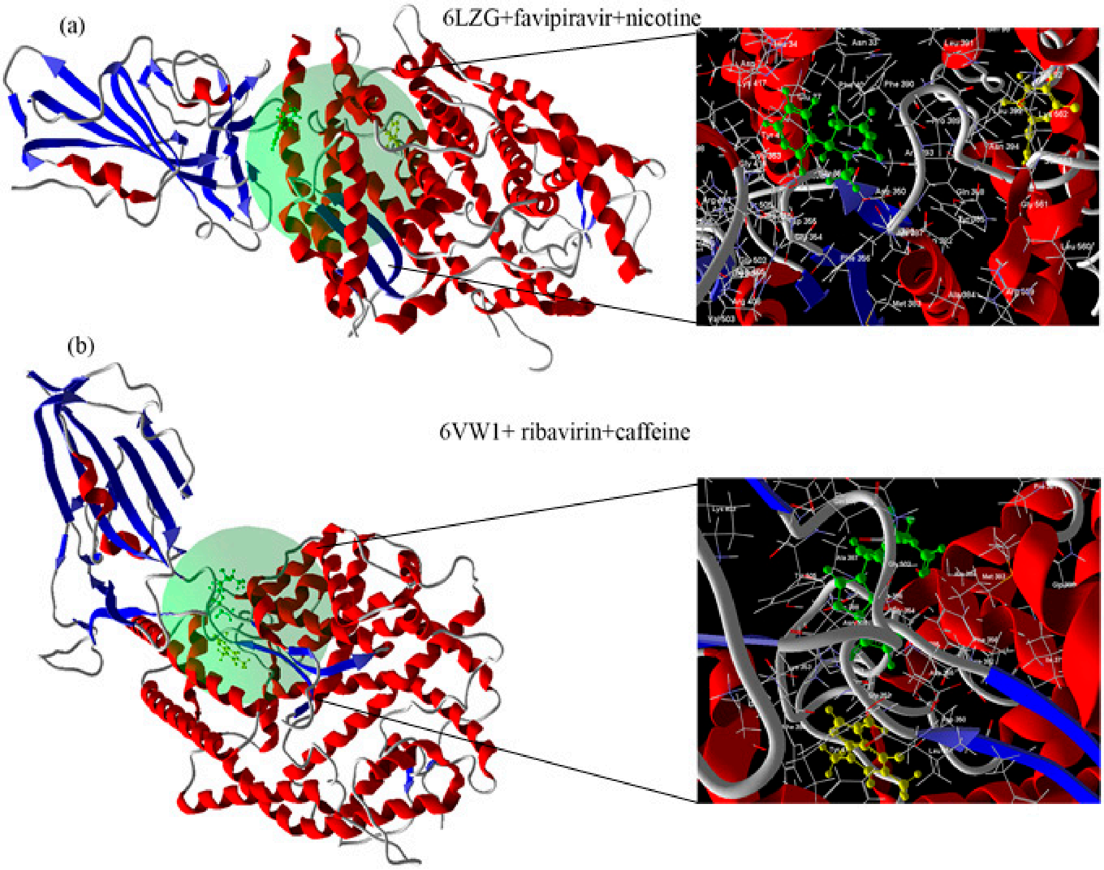

Figure S1. Interaction between ACE2-RBD/CTD (6VW1, 6LZG) complex with **(a)** 6LZG+favipiravir+nicotine and **(b)** 6VW1+ribavirin+caffeine that green region and magnified inlays represent active sites.

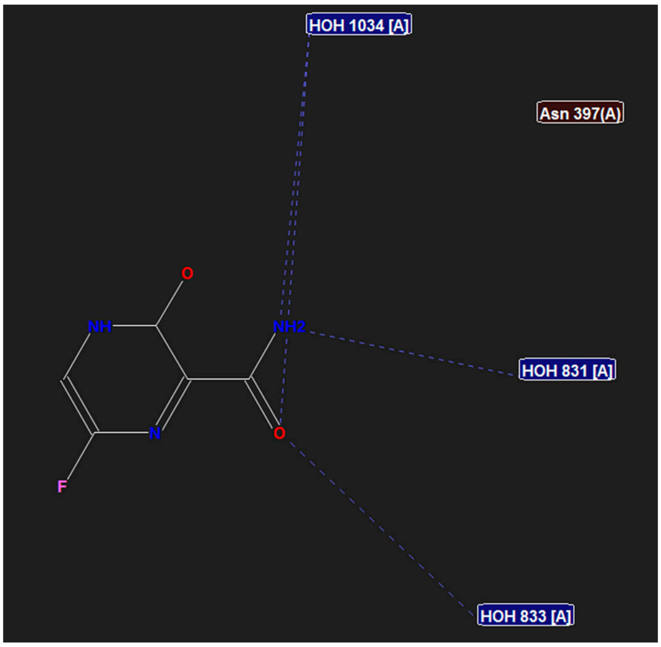

Figure S2. 2D representation of favipiravir binding mode with receptor binding site of SARS-CoV-2 S protein (6LZG+favipiravir+nicotine).

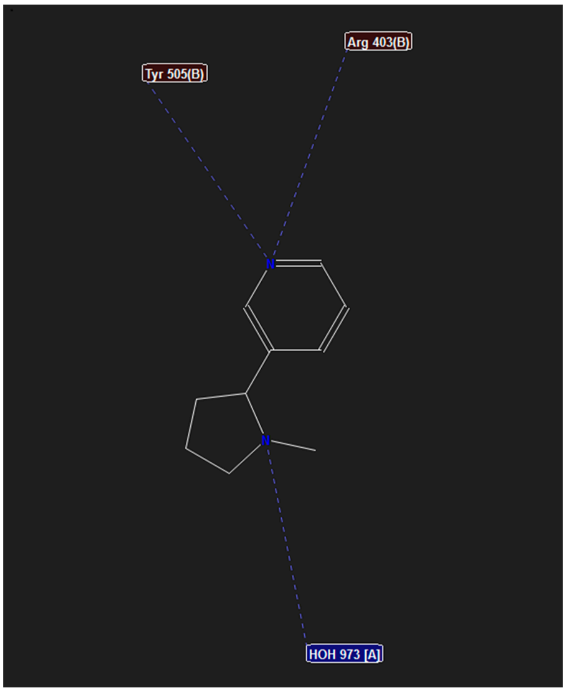

Figure S3. 2D representation of nicotine binding mode with receptor binding site of SARS-CoV-2 S protein (6LZG+favipiravir+nicotine).

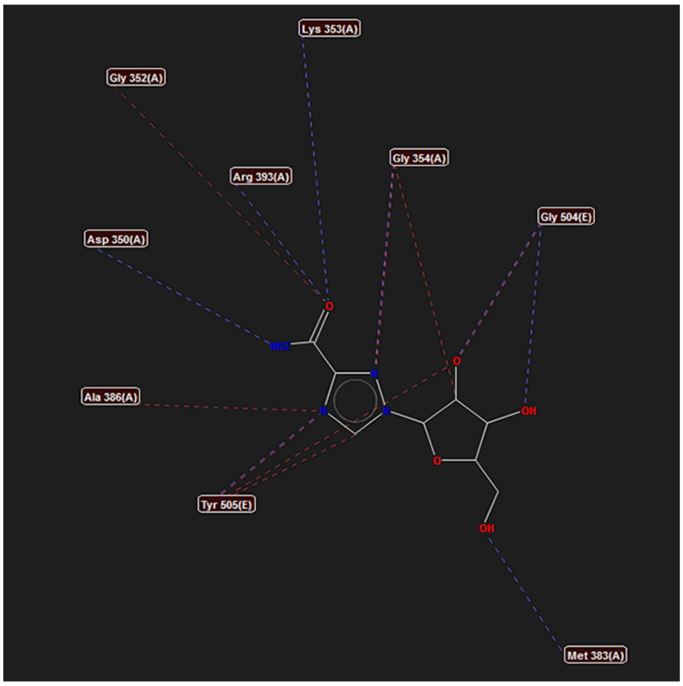

Figure S4. 2D representation of ribavirin binding mode with receptor binding site of SARS-CoV-2 S protein (6VW1+ ribavirin+caffeine).

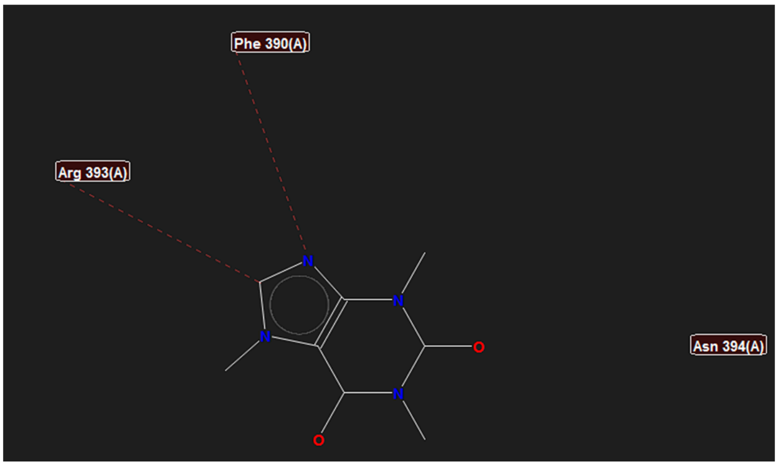

Figure S5. 2D representation of caffeine binding mode with receptor binding site of SARS-CoV-2 S protein (6VW1+ ribavirin+caffeine).
